# An analytical study of employee loyalty and corporate culture satisfaction assessment based on sentiment analysis

**DOI:** 10.3389/fpsyg.2022.971569

**Published:** 2022-11-01

**Authors:** Jie Xie, Ri Le Ge Su, Jaehoon Song

**Affiliations:** Woosuk University, Jeollabuk-do, South Korea

**Keywords:** business, employee loyalty, corporate culture satisfaction assessment, sentiment analysis, association rules

## Abstract

As an important factor related to the interests of enterprises, the attitude and behavior of employees are related to the company’s survival and the realization of business objectives. However, in recent years, with the rapid development of emerging industries and industry changes, the turnover rate of employees in the whole enterprise has been greatly improved. Frequent turnover of personnel will have a great impact on the stability of the company, the competitiveness of the enterprise, and the operating cost. Employee loyalty and corporate culture satisfaction assessment is the core of sustainable corporate development, but most of the traditional analysis studies are based on the performance approach, using employee overtime, employee achievement, and company effectiveness as data sources, the former performance data is only a side view of the company’s situation, the latter as the data source of this study is a one-sided and involute form, and the data is not stable. Therefore, this paper proposes an analysis of employee loyalty and corporate culture satisfaction assessment based on sentiment analysis, using association rules and sentiment lexicons from anonymous employee evaluations to analyze the actual data.

## Introduction

Since ancient times, whether at the national level or at the level of individual organizations, those who win people will prosper, and those who lose people will die. The competition between building construction enterprises, there are brands, technology, image reputation, economic strength, etc., but in the final analysis, is the competition of talents. Comrade Xi Jinping, President of the People’s Republic of China, pointed out during his visit to Shenyang Machine Tool Group that talent is the most valuable asset, and that we should do our best to cultivate and make good use of it, and increase investment in research and development. When young people grow up, there is hope for the enterprise and hope for the country (Ahmad and Laroche, 2016).

Staff are the backbone of the company and are the company’s sustainable development guarantee. Overtime, all areas of business have followed suit, creating a wide stage for the selection of talent. However, due to technical difficulties, the skills of employees have become increasingly important, and the company and employees take what they need (Bengio et al., 2003).

At present, a number of new situations have emerged among the employees of an enterprise, highlighted by the fact that “it is difficult to recruit talents” and “it is difficult to retain talents.”

Since the large-scale introduction of graduates from an enterprise in 2005, the enterprise has been facing all higher education institutions nationwide to select and recruit talents, but the recruitment results are not optimistic, with very few graduates from famous universities working for the enterprise. Even though many graduates from “985” and “211” institutions were recruited, some of them eventually chose to breach their contracts or resign when they were about to embark or take up their jobs. A recent attrition figure from the enterprise shows that over the past 5 years, a total of 2,673 people have left the company’s 16 subsidiaries, including 2,363 technical cadres, an average annual loss of 473 technical cadres. Although this loss is a tolerable figure for a large state-owned enterprise, a careful analysis shows that the lost employees are mainly young technical cadres who have graduated from full-time universities and colleges, are engaged in engineering professions, and have certain working experience (Chatterjee, 2006). The degree of attrition is increasing year by year. More importantly, in a market-oriented environment, employees in state-owned enterprises lose a large number of employees to competing companies in the market through employee poaching. For a long time, many managers attach importance to the use of employees, while ignoring the employees’ satisfaction, which affected the morale of the employees, at the same time increasing the cost of the introduction and training of talents, and claim to the leadership, the owner of the travel non-stop, attaches great importance to, but for the negligence of the employee life feelings and needs concern and care, often ignored the employee satisfaction. This affects employee morale and increases the cost of bringing in and developing talent (Chen and Xie, 2008).

## Technical analysis

### Corporate culture satisfaction research

As early as the beginning of the 20th century, Keith (Keith) and others stressed that economic activities should meet the needs and aspirations of employees, but research on employee needs and employee satisfaction has been going on for the last 20–30 years. In the 1960s, a definition of employee satisfaction emerged abroad, but no theoretical system of employee satisfaction was developed at that time. In the late 70s and early 80s, Oliver, Olson, and other scholars proposed the “expectation unconfirmed” model, which considered employee expectations as a measure of employee satisfaction. From the 1980s to the 1990s, many scholars such as Ernest (Cheng and Ho, 2015), Robert, and Tse further extended and supplemented this model from the perspective of psychology and management, but they only paid attention to the influence of expectations on satisfaction, but neglected to study the basic determinants of satisfaction – needs. In the mid-1990s, Spreng, Mackenzie, and others in the USA continued to refine the theory by introducing the factor of desire into the old model, arguing that when employees compare their perceptions of product or service performance with their desires and expectations Much of the research in employee satisfaction theory in the late 1990s focused on exploring the relationship between employee satisfaction, employee satisfaction, employee loyalty and corporate profits (Chua and Banerjee, 2015).

From the above series of research processes, most of the foreign scholars’ research content is still focused on the theoretical model of employee satisfaction and the relationship between employee satisfaction and employee behavior, with emphasis on the composition of the elements influencing employee satisfaction and the importance of employee satisfaction to the enterprise. In terms of research methods, there are qualitative and quantitative studies, but there is no unified method for measuring employee satisfaction, and the system is mainly constructed according to different industries and enterprises (Fang et al., 2016). In terms of the application of employee satisfaction, in 1986 a market research company in the USA published its first ranking of consumer satisfaction in the automotive industry using employee satisfaction as a criterion. Soon after, the employee satisfaction theory was introduced into Japan and quickly swept through the Japanese corporate world. With the intensification of market competition and the gradual improvement of employee satisfaction theory, more and more enterprises have introduced an employee satisfaction strategy, which has become a competitive tool for enterprises. In 1989, Sweden was the first in the world to apply the employee satisfaction theory index, referred to as SCSB, on a national scale, to monitor the operation of the national economy. In 1994, the USA also launched a nationwide employee satisfaction index (ACSI), and China carried out research work on the China National Customer Satisfaction Index (CCSI) in 1998, and in July 2002 the basic framework and operational plan of the CCSI evaluation system had basically taken shape, and the conditions for the timely establishment of the CCSI evaluation system were basically in place. China’s research on employee satisfaction theory is still in its infancy, in theory, it mainly follows the trend of foreign theoretical development, in practice, it is only limited to a few joint ventures or foreign-funded units, and there is a large gap between the theoretical research and practical application of employee satisfaction abroad. At present, the research and application of employee satisfaction in China are more prominent in China Quality Association, Shanghai Quality Association, etc. In particular, the Employee Evaluation Centre of the Shanghai Institute of Quality Management Science is in the leading position in China in terms of research and application of CS. In 1999, it conducted a joint assessment of the satisfaction index of Shanghai taxi employees with the Shanghai Taxi Administration, which was the first time in China that the satisfaction of a region and an industry was measured, and this assessment also achieved very good results (Ghose and Ipeirotis, 2011).

### Employee loyalty research

The idea and practice of fostering employee loyalty emerged early in business, but it was not until the mid-1990s that it became a trend that caught on quickly among companies. American scholars Slater and Marver found that it costs four to six times more to attract a new employee than to retain an old one; that a 2% reduction in employee turnover equates to a 10% reduction in costs; and that for most companies if they can maintain a 2% growth rate in employee loyalty, their profits can almost double within 5 years (Guo and Zhou, 2016).

#### Foreign research

According to Buchanan, employee loyalty is the dependence of employees on the company. According to Mowday et al. (1982), employee loyalty means that employees take the company’s culture as their work criteria, always keep in mind the company’s culture, highly affirm the company’s culture and goals, and are willing to make contributions to the company’s development and stick to their work. Voyles et al. (2001) points out that employee behavior is less likely to change in the long run than attitude, so employee loyalty should be reflected by employee behavior.

#### Domestic research

Chinese scholars began to study employee loyalty in the 1990s. At the beginning, they focused on foreign academic achievements. On the basis of drawing lessons from foreign scholars and combining them with my own research findings, this paper explains employee loyalty from a new perspective. In 2000, Aning (2000) proposed that the loyalty of employees is stratified, that is, there are high loyalty and low loyalty. Yan (2001) believes that employee loyalty is a kind of behavior in which employees work hard and do their best to achieve the strategic goals of the company.

In his 1985 book Relationship Marketing, Ben Jackson pointed out the importance of building long-term relationships with employees, followed by a large body of research that empirically justified the idea. Done Schults proposed the 4Rs marketing theory (relevance, reflection, relationship, reward) based on the concept of employee loyalty, aiming to use the 4Rs to strengthen the relationship with employees and increase loyalty. Frederick Reichheld, a consultant at Bain & Company, uses a number of case studies from leading loyalty management companies to propose how to design business systems based on the principles of value and loyalty, in an attempt to use the integration of information technology and marketing theory to build relationships with employees and increase employee loyalty.

The measurement of employee loyalty is in fact a quantitative description of employee loyalty. Scholars at home and abroad have proposed different measurement models, such as Blackwell’s value-loyalty model, McDougall’s value-loyalty model for the service industry, Ryan’s loyalty model for energy service organizations (Hai et al., 2015) and domestic, the value satisfaction- loyalty model of Wang Yuexing (Hong et al., 2017) and the employee loyalty model proposed by Chen Mingliang (Hu and Liu, 2004). In addition, although the ASCI model in the USA and the SCSI and ECSI models in Sweden are used to measure employee satisfaction indices, the ultimate aim of these models is to predict and explain employee loyalty.

Many scholars are conducting research on employee loyalty, but research on this topic is still immature and many issues are still in the exploratory stage. In particular, a range of issues such as the measurement of employee loyalty and the patterns of change in employee loyalty are being studied in depth (Huang et al., 2015).

## Research methodology design

### Feature lexicon construction

Different employees use different words when commenting on a company, even if they are commenting on the same feature. For example, “the company’s welfare is good today” and “the company is really great today,” both of which describe the same features. Therefore, in this paper, the initial feature lexicon is constructed based on the merging of synonyms in order to reduce the dimensionality of the feature lexicon (Jiao et al., 2007).

Secondly, the granularity of the extracted features is not fine enough. For example, in the following comments “corporate culture is good” and “corporate culture is worth learning” (Ansoff, 1965), the former is the evaluation of corporate culture in general, while the latter is the culture of corporate culture itself, but most of the studies, when extracting employee evaluation features, attribute the above comments attributed to the evaluation of the enterprise, this paper gets feature-attribute two-layer structure in feature extraction, such as odd enterprise-culture, in order to obtain user needs in a more fine-grained manner (Davis, 1960).

**FIGURE 1:**
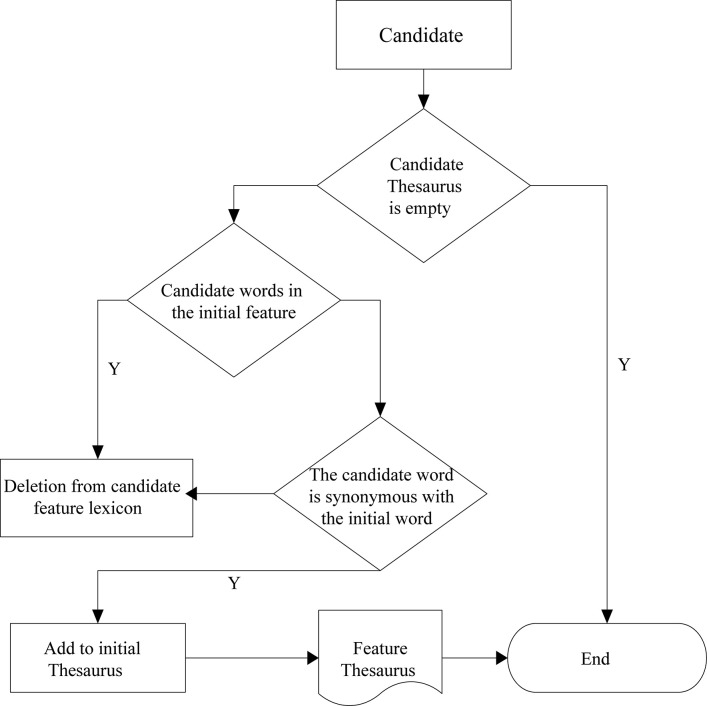
Flowchart.

Therefore, the construction of the feature lexicon in this paper has the following two purposes: (1) to reduce the feature dimension of employee reviews by merging and replacing synonyms based on word similarity analysis; (2) to establish feature-attribute subordination relationships between features. The specific construction process is as follows, as shown in Figure 1.

1. Create an initial feature dictionary. Based on the detailed information of enterprises, it classifies them, including the attribute relations of features, and constructs an initial feature dictionary.
2. Screen the candidate feature words. In the evaluation of employees, nouns or noun phrases are generally used to express, so this paper starts from Chinese word segmentation and part-of-speech tagging to eliminate inactive words and select some nouns and noun groups from them.
3. Similarity of words. Based on the corpus, Word2vc similarity model is used to analyze the synonymy relation, and the feature words are synthesized from the original feature words.

### Sentiment lexicon construction

Sentiment dictionaries are crucial for sentiment analysis in text mining, but the current public sentiment dictionary is too slow to be updated, while the development of online language is extremely fast. Therefore, this paper extends the sentiment lexicon on the basis of the public sentiment lexicon with the help of word similarity analysis to form a sentiment lexicon. Its construction process is the same as the feature lexicon construction process, and its construction process is as follows (Sethi, 1975):

1. Create an initial feature dictionary. The “elementary” dictionary is composed of three parts, namely “THE Knowledge Network Dictionary,” “NTUSD,” and “College Emotion Dictionary.”
2. Screen the candidate feature words. In the process of word segmentation, annotation, and deactivation in Chinese, people often choose adjectives as candidates.
3. Similarity of words. Like the constructional feature dictionary, the original emotion dictionary was derived from similarity analysis and the artificial addition of words.

In this paper, 19,446 sentiment words, 7,350 positive sentiment words, and 12,096 negative sentiment words were obtained from the corpus-based sentiment lexicon construction. Some of the results are shown in Table 1.

**TABLE 1:** Example of a sentiment lexicon.

Emotional words	Emotional value	Emotional words	Emotional value
A	1	E	–1
B	1	F	–1
C	1	G	–1
D	1	H	–1

### Association rule mining

Two or more variables are said to be associated with each other if there is some pattern between their values. Association rules refer to the correlation between different items that occur in the same event.

The mining of association rules in a database can be formally defined as:

Let *I* {*i*1, *i*2,…, *in*} be the set of all terms, *D* is the set of all transactions and each transaction *T* is the set of some items, *T* {*t*1, *t* 2,…, *tn*}, *ti I*, and T is uniquely identified by an identifier called *TID*. *X*_‵_*Y* is the set of data items and *K* is the items in *X.* It is called the K-data item set. The association rule in D is represented as the association equation, and *X I*, *Y I*, *X Y*, X is called the premise or left-hand part (LHS) and Y is the successor or right-hand part (RHS). In general, the association rule can be expressed as *X*1 *X*2 *Xn Y*1 *Y*2… *Yn*_°_.

It is governed by both support, which indicates the frequency of the rule, and confidence, which indicates the strength of the rule. The support of a rule X Y in D is the ratio of the number of items in the data item set containing both X and Y to the number of items in all data items, support (*X Y*)*T*:*X Y T*,*T D D.*

Given a data item set D, the association rule mining problem is to generate association rules with support and confidence greater than the user-given minimum support (min-supp) and minimum confidence (min-conf), respectively. A rule is considered valid only when its support and confidence are greater than m in-supp and min-conf, respectively, and is called a strong association rule. When the support of X is greater than min-supp, X is said to be the set of data items of high frequency.

The association model essentially describes the closeness or relationship between a set of data items. Association rules can also be classified into Boolean association rules and quantitative association rules, categorized by the type of data dealt with in the association rule.

### Apriori algorithm

The Apriori algorithm was proposed by Agrawal and Srikant in 1994 and is one of the classic algorithms for data mining. Based on the property of Apriori that all non-empty subsets of a frequent itemset must also be frequent itemsets, the algorithm first generates an i-order frequent itemset based on the i – 1 frequent itemset and then scans the dataset to determine the i-order frequent itemset. The initial traversal of the database by the Apriori algorithm is to calculate only the number of specific values for each item to determine the large 1-item set. The i-th traversal (i > 1) consists of the following two stages.

**Table:** 

1. using the large set of items Li – 1 found in the i –1 traversal and the *Apriori*-gen function to generate the candidate set Ci; and 2. Scan the database and calculate the support of Ci. The Apriori algorithm is described as follows. L=find_frequent_1-itemsets(D);l/All frequent 1-item sets found for (i=2: L;_‵_.≠;i++) { C;=apriori_gen(L;.,minsupp);ll Generating candidate sets for each transaction t D;//Scan all transactions { C=subset(Ci,t);//Identify the set of all candidates belonging to t for each candidate c C c.count++;l/Support count increments of 1 } Li={cCk c.countminsupp};/ Extracting frequent i-item sets } return L=UkLk;l/

## Analysis of research findings

### Sentiment analysis

(1) Sentiment analysis and weighting process

First, the emotion dictionary and the initial score of the feature word are combined to get the four-dimensional “feature,” “attribute,” “emotion word,” “attribute,” and “emotion word.” After considering the function of adverbs of degree, the author weights the polarity of mood words with adverbs of degree and divides them into four grades, as shown in Table 2.

**TABLE 2:** Polarity values for degree adverbs.

Degree adverb	Polarity value
Too, very, completely, incomparably, most	2
Slightly, slightly, more……	1.5
Slight, relative, some……	0.5
Positive emotion words without degree adverbs	1
Negative emotion words without degree adverbs	−1

In this paper, the sentiment analysis results are weighted according to the polarity value of the degree adverb, and the initial sentiment score is weighted according to the polarity value of the degree adverb for sentiment words modified by degree adverbs, but not for sentiment words not modified by degree adverbs.

Sentiment score = initial score, without degree adverb modifier

The sentiment values are weighted to give a six-dimensional array of < feature, attribute, sentiment word, initial score, degree adverb, sentiment value >. As online reviews have very high positive ratings, in order to balance the impact of higher ratings on the final score, the sentiment mean of each feature is calculated using positive and negative sentiment means, and the corrected sentiment score is calculated as follows.

*Score* = *postive* + *negtive*

Where postive and negtive represent positive and negative sentiment averages, respectively. The final sentiment analysis results were obtained after degree adverb weighting and summary analysis < feature, attribute, number of evaluations, positive evaluation ratio, negative evaluation ratio, initial score, sentiment value >.

### Employee loyalty analysis

The four drivers of loyalty can be considered as the psychological factors that lead to employee loyalty or not, and they work together with the objective environment to determine the loyalty behavior of employees. Their loyalty to the need to translate into actual behavior for the enterprise to have real significance, after all, the profit is obtained through the efforts of employees. However, it is also one-sided to determine the loyalty level of employees only on the basis of performance and other behaviors, because actual factors such as traffic and competition can also lead to repeated consumption at low levels of emotional loyalty, so we should combine emotional and behavioral loyalty when building models to evaluate employee loyalty. Only in this way can loyalty be evaluated comprehensively and accurately.

In this study, 2,325 employees of a company were selected to carry out this loyalty analysis, and the following data were obtained after each person submitted an evaluation of the company. The score is shown in Table 3.

**TABLE 3:** Score table.

Name	Number of people	Number of points
Loyalty	200	9.0
Loyalty	1300	7.0
Loyalty	500	6.0
Loyalty	320	5.5
Loyalty	25	4.5

**FIGURE 2:**
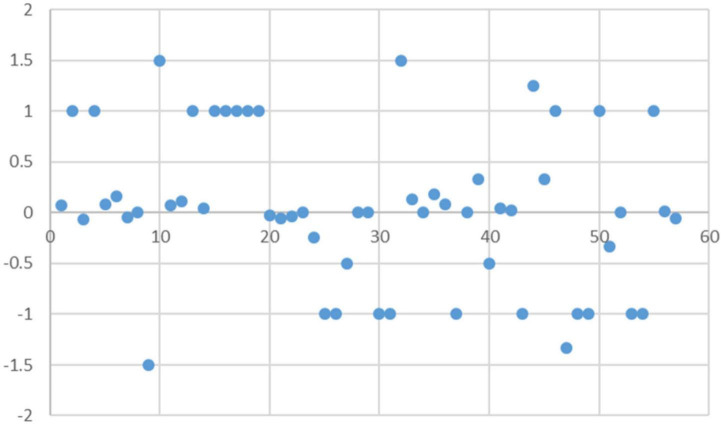
Distribution of affective values.

In the above step, the sentiment value corresponding to each < feature, attribute, sentiment word > was obtained through the degree adverb weighting process. In order to obtain the user’s evaluation of different product features, the sentiment score of each product feature was calculated according to the formula in order to aggregate the evaluation opinions of all users. The sentiment analysis results were summarized and analyzed as shown in Figure 2. The sentiment values were distributed in the [−2, 2] interval, with a relatively even distribution of employee sentiment and no significant differences.

### Analysis of corporate culture satisfaction assessment

In order to improve the satisfaction of the corporate culture, enterprises must first establish a people-oriented management concept and use this concept to create a different corporate culture. When enterprises respect employees, treat them sincerely, and put themselves in their shoes, employees feel that they are a member of the company and the master of the company, and actively create a harmonious relationship between superiors and subordinates, and between employees. Secondly, to stimulate the passion of the staff through the incentive mechanism, to explore the potential of the staff, high-tech enterprises in high-skill labor, the need for employees to think deeply and rigorous argumentation, in order to create work results. The opposite of an incentive mechanism is a restraint mechanism: to be without a restraint mechanism is like an engine lacking a braking device, and the development of employees will go astray.

The object of this study is 2,325 employees of a company, and they are asked to conduct a survey on their satisfaction with corporate culture. Table 4 shows the data obtained after each person has submitted an evaluation of corporate culture.

**TABLE 4:** Score table.

Name	Number of people	Number of points
Corporate culture satisfaction	100	9.8
Corporate culture satisfaction	250	8.5
Corporate culture satisfaction	400	8.3
Corporate culture satisfaction	525	7.9
Corporate culture satisfaction	1050	> 7

**FIGURE 3:**
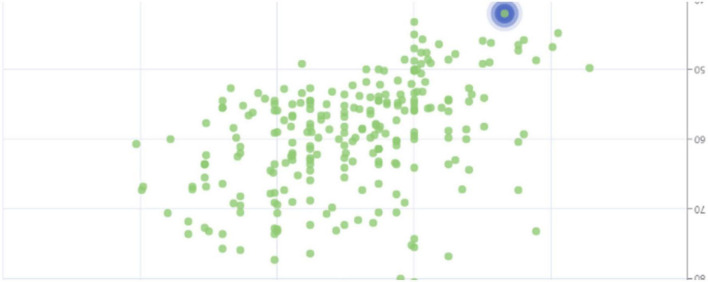
Distribution of sentiment values.

In the above step, the sentiment value corresponding to each < feature, attribute, sentiment word > was obtained by the degree adverb weighting process. In order to obtain the user’s evaluation of different product features, the sentiment score of each product feature was calculated according to the formula in order to aggregate the evaluation opinions of all users. The sentiment analysis results were summarized and analyzed as shown in Figure 3. The sentiment values were distributed in the interval [0, 2] and the corporate culture satisfaction analyses were all positive, indicating a high level of recognition of this corporate culture.

## Conclusion

This research on the evaluation and analysis of employee loyalty and corporate culture satisfaction based on sentiment analysis starts from the objective anonymous evaluation of employees, the construction of the sentiment lexicon and the construction of the feature lexicon, then the data analysis of employees’ evaluation, the extraction of the corresponding scores and the construction of specific process steps.

An effective method of implicit feature extraction and implicit emotion analysis is established. Based on the existing research on implicit characteristics, this paper proposes a method of implicit characteristics and implicit emotion analysis that combines the rule base of emotional characteristics with the similarity of utterance, which makes up the gap in the field of implicit emotion analysis. On the basis of this, we use the text mining method based on sentiment analysis to analyze the anonymous objective evaluation of employees. With the traditional technique of data mining technology, mental models, compared to using the sentiment analysis technology can quickly and accurately obtain employee loyalty and satisfaction with the enterprise culture. This can save costs for the enterprise, and can improve the analysis of the timeliness and accuracy and put forward suggestions for the design of the product and improve. In today’s e-commerce information overload, this has important practical significance.

## Data availability statement

The raw data supporting the conclusions of this article will be made available by the authors, without undue reservation.

## Author contributions

JX was experimental designer and executor of the experimental study, completed the data analysis, and wrote the first draft of the manuscript. RS was conceptualizer and the person in charge of the project, directing the experimental design, data analysis, and thesis writing and revision. JS was overall planner and supervisor of the study and the manuscript, and is responsible for the communication and payment of publication fees during the submission and publication process. All authors contributed to the article and approved the submitted version.
